# A pilot randomised controlled trial to assess the feasibility and acceptability of recovery-focused therapy for older adults with bipolar disorder

**DOI:** 10.1192/bjo.2022.582

**Published:** 2022-10-24

**Authors:** Elizabeth Tyler, Fiona Lobban, Christopher Sutton, Bogdan Hadarag, Sheri Johnson, Colin Depp, Deborah Duncan, Steven H. Jones

**Affiliations:** Spectrum Centre for Mental Health Research, Division of Health Research, Lancaster University, UK; Division of Population Health, Health Services Research & Primary Care, University of Manchester, UK; Division of Health Research, Lancaster University, UK; Department of Psychology, University of California, Berkeley, USA; Department of Psychiatry, UC San Diego School of Medicine, University of California, San Diego, USA

**Keywords:** Bipolar affective disorders, individual psychotherapy, psychosocial interventions, randomised controlled trial, patients

## Abstract

**Background:**

Despite increasing evidence for the effectiveness of individual psychological interventions for bipolar disorder, research on older adults is lacking. We report the first randomised controlled trial of psychological therapy designed specifically for older adults with bipolar disorder.

**Aims:**

To evaluate the feasibility and acceptability of recovery-focused therapy, designed in collaboration with older people living with bipolar disorder.

**Method:**

A parallel, two-armed, randomised controlled trial comparing treatment as usual with up to 14 sessions of recovery-focused therapy plus treatment as usual, for older adults with bipolar disorder.

**Results:**

Thirty-nine participants (67% female, mean age 67 years) were recruited over a 17-month period. Feasibility and acceptability of recruitment, retention (>80% observer-rated outcomes at both 24 and 48 weeks) and intervention processes were demonstrated. The majority of participants started therapy when offered, adhered to the intervention (68% attended all sessions and 89% attended six or more sessions) and reported positive benefits. Clinical assessment measures provide evidence of a signal for effectiveness on a range of outcomes including mood symptoms, time to relapse and functioning. No trial-related serious adverse events were identified.

**Conclusions:**

Recovery-focused therapy is feasible, acceptable and has the potential to improve a range of outcomes for people living with bipolar disorder in later life. A large-scale trial is warranted to provide a reliable estimate of its clinical and cost-effectiveness.

Approximately 0.5–1% of older adults live with bipolar disorder,^1^ which will place significant and increasing demands on healthcare services. The UK National Institute for Health and Care Excellence (NICE) bipolar disorder clinical guideline^2^ states that older people with bipolar disorder should be offered the same range of treatments as younger adults. Such treatments may not be appropriate because of significant differences in the nature of bipolar disorder in later life. Bipolar disorder in older adults differs in presentation, is more complex, and is accompanied by high rates of physical comorbidities^3^ and poorer cognitive function even during euthymia,^4^ which may significantly affect psychosocial outcomes.^5^ As cognitive functioning declines, older adults with bipolar disorder may struggle to apply effective coping responses to difficult situations,^4^ including adopting more passive coping styles than non-clinical older adults.^6^ Despite these differences, few studies have evaluated psychological interventions developed for older people with bipolar disorder. In the USA, a psychosocial skills training programme for older people with various mental health problems (schizophrenia, schizoaffective disorder, depression and bipolar disorder) improved community living skills, functioning and self-efficacy, and reduced psychiatric symptoms.^7^ Additionally, medication adherence skills training led to improvements in medication adherence and management, depressive symptoms and quality of life for older people with bipolar disorder.^8^ However, it is not clear from the first study what the outcomes are specifically for older people with bipolar disorder and neither study targeted personal recovery, which is highlighted in national policy^9^ and NICE guidelines.^2^

Over the past four decades, the personal recovery movement, led by patients, has called for a new approach to define ‘recovery’. This has led to a shift from recovery outcomes being based predominantly on eradicating symptoms and ‘cure’, and a move toward building strength and resilience in an individual, enabling them to take control of their life and mental health problems. This paper reports on a recovery-focused therapy (RfT) intervention for bipolar disorder, adapted to meet the needs of older adults (RfT-OA). Research for working-age adults has shown this approach is beneficial in terms of personal recovery and relapse outcomes.^10^ This is the first study to evaluate the feasibility and acceptability of RfT for older people living with bipolar disorder.

## Method

The authors assert that all procedures contributing to this work comply with the ethical standards of the relevant national and institutional committees on human experimentation and with the Helsinki Declaration of 1975, as revised in 2008. All procedures were approved by the UK National Health Service (NHS) Ethics North West – Preston Committee process (reference 15/NW/0330). Written informed consent was provided by all participants. The study was preregistered with the ISRCTN registry (identifier ISRCTN13875321), and a study protocol informed by the Standard Protocol Items: Recommendations for Interventional Trials (SPIRIT) Guidelines^11^ was pre-published.^12^ The study is consistent with the Consolidated Standards of Reporting Trials (CONSORT) extension for pilot and feasibility trials^13^ (see Supplementary File 1 available at https://doi.org/10.1192/bjo.2022.582).

Three study changes were necessary after protocol publication. Minimum age was reduced from >65 to ≥60 based on the United Nations^14^ definition of older adults and in line with best practice.^15^ The latest version of the Structured Clinical Interview for DSM-5, research version (SCID-5-RV^16^) was used. Outcome assessments were not conducted blind to allocation because of resource constraints.

### Objectives

The study aim was to evaluate the feasibility and acceptability of RfT-OA in an randomised controlled trial design. The first objective was to determine the feasibility of RfT-OA in terms of whether clinicians would refer older adults into a randomised controlled trial; whether older adults would self-refer into a randomised controlled trial; whether older adults with bipolar disorder would consent to participate in an randomised controlled trial of a psychological intervention offered in addition to treatment as usual (TAU) versus TAU alone; and participant attrition rates (overall and by study arm) during assessment, intervention and follow-up. The second objective was to determine the acceptability of RfT-OA in terms of whether individuals adhered to the intervention and participants’ experiences of the intervention. Finally, with regards to future research, we aimed to identify the most appropriate primary outcome measure and estimate parameters needed to determine the sample size for a future trial.

### Trial design

This was a parallel, two-armed, randomised controlled trial comparing TAU with up to 14 sessions of RfT-OA in addition to TAU, conducted across two NHS trusts in North-West England. A nested qualitative study explored the acceptability of RfT-OA.

Participants were allocated to trial arms with simple (1:1) randomisation by independent researchers at Lancashire Clinical Trials Unit, using the ‘Sealed Envelope’ randomisation programme (www.sealedenvelope.com), after baseline assessment. The study was overseen by a trial steering committee and a service user reference group.

### Participants

A target of 50 participants (25 per treatment arm) was considered sufficient to obtain robust feasibility and acceptability information about trial procedures and the RfT-OA therapy, and to allow for expected attrition rates.^17^ Participants were recruited from NHS mental health services, voluntary groups, advertisements in local media and on the Bipolar UK website (https://www.bipolaruk.org), and from ‘Spectrum Connect’, a confidential database (maintained by a research team at Lancaster University) of individuals who have previously consented to being approached about research studies. Recruitment was from December 2016 to January 2018, and May 2019 to September 2019; final follow-up was in September 2020 because of the lead investigator's maternity leave. Interested individuals were screened for provisional eligibility with the Mood Disorder Questionnaire (MDQ^18^). Participants aged ≥60 years from North-West England who screened positive on the MDQ were invited to a baseline interview.

### Eligibility interview

Written informed consent was obtained from all participants. They were assessed with the SCID-5-RV and the Montreal Cognitive Assessment (MOCA^19^) to confirm eligibility criteria, and completed the observer-rated and self-report measures (see ‘Clinical and functional outcomes’ section).

#### Inclusion/exclusion criteria

Inclusion criteria were as follows: aged ≥60 years; SCID-5-RV diagnosis of bipolar disorder (type 1 or 2); no current episode of mania, hypomania, depression or mixed episode in the past 4 weeks; and sufficient English-language fluency to consent and take part. Exclusion criteria were an MOCA score of ≤22 or currently receiving psychological therapy.

### RfT-OA

The original therapy manual was co-produced with adults with lived experience of bipolar disorder,^10^ and adapted for this study with older adults (RfT-OA). RfT focuses on helping clients to identify meaningful, personal goals to help them live well alongside their bipolar disorder experiences. These goals are not predetermined to have a clinical focus because the individual with bipolar disorder is assumed to be best placed to determine their own priorities. As such, personally defined goals can be symptom-related or focus on other areas such as relationships, social engagement or work. During the initial sessions, the therapist and client develop a shared understanding of recovery and how working toward goals that are of personal value may have a significant impact on the person's life. Developing an idiosyncratic formulation is a fundamental part of the process, ensuring the therapy approach is consistent with the person's needs. RfT highlights the importance of developing and maintaining a flexible approach to engagement during therapy, placing emphasis on building rapport and the timing, duration and frequency of sessions.

Adaptations for RfT-OA were informed by a literature review on adapting psychological interventions for older adults with mental health problems,^20^ and three focus groups with older people with bipolar disorder.^21^ Adaptations included building confidence, competence and assertiveness; greater emphasis on relationship with the therapist; allocating more time to explore extensive and complex histories; and explicit acknowledgement of the wealth of experience the older person with bipolar disorder is bringing to the therapeutic relationship. Specific adaptations to enhance memory and learning included repetition and using session summaries, consistent with existing literature.^20^ Participants were offered 14 therapy sessions, with six or more considered an appropriate threshold for the therapeutic dose, based on the original RfT study.^10^

### TAU

No changes were made to any other interventions. Psychiatric medication use and previous therapy experience were recorded.

### Therapists

RfT-OA was delivered by three qualified clinical psychologists. E.T. delivered the majority of the therapy (*n* = 17). F.L. and S.H.J. worked with one client each. All were trained in the use of RfT-OA and attended fortnightly peer supervision.

### Key outcomes: feasibility and acceptability

Information was collected on number of referrals per month, recruitment sources, number of people assessed for eligibility and provided consent. Participant retention was assessed during assessment, intervention and follow-up periods, including completion of outcome measures. Prespecified criteria were used to interpret the findings in terms of feasibility and acceptability outcomes for progression to a definitive randomised controlled trial, using a traffic light system.^22^

### Additional outcomes: feasibility and acceptability

Therapeutic alliance was assessed at the start (session three or four), middle (session eight or nine) and end of therapy (session 13 or 14), using the Working Alliance Inventory – Short Form (therapist and client forms).^23^

Clients also rated the therapy on two scales: how useful they found the therapy (from 0 (not at all) to 10 (extremely)) and whether they would recommend the therapy to a friend experiencing similar problems (from 0 (definitely not) to 10 (definitely yes)). Participants were given a copy of the scales and an envelope during their penultimate therapy session, to return at the final session.

### Clinical and functional outcomes

Multiple clinical and functional outcome measures were used to assess participant attrition rates, participant views of measures from qualitative interviews and to provide preliminary data on outcomes. Participants were followed from baseline, with telephone interviews at 12, 24, 36 and 48 weeks (observer-rated measures), and by post at 24 and 48 weeks (self-report measures). The full list of observer and self-report measures can be found in Supplementary File 2.

The candidates for primary outcome measure were the Hamilton Rating Scale for Depression (HRSD^24^), Bech–Rafaelsen Mania Scale (MAS^25^), modified Longitudinal Interval Follow-Up Evaluation (SCID-LIFE^26^) to assess time to relapse, and the Bipolar Recovery Questionnaire (BRQ^27^), based on the original RfT study.^10^

### Quality assurance

D.D. completed 18 telephone follow-up assessments when E.T. was on maternity leave. Interrater reliability between E.T. and D.D. was assessed at both baseline and follow-up.

### Data analysis

Analyses were conducted with SPSS for Windows version 25 (IBM Corp.^28^), and followed a prespecified statistical analysis plan, approved by the trial steering committee.

Analysis was ‘as randomised’. Summary statistics were used to estimate key parameters, such as rates of recruitment, consent to the trial and retention to therapy and follow-up assessments. Linear models were used to assess the effect of RfT-OA versus TAU alone on each continuous outcome measure, with baseline value of the relevant measure as covariate. Time to first relapse was analysed with Kaplan–Meier plots and the Cox regression modelling, with time since last episode as the covariate. Separate analyses were performed for three types of relapse (any episode, depressive or manic). The focus of the analysis was point estimation with 95% confidence intervals, rather than statistical significance. For statistical models, missing data were assumed to be missing at random (dependent only on intervention group and baseline).

Pooled standard deviations for each continuous outcome, and median time to relapse, were used to estimate key parameters needed (in conjunction with data from other relevant trials, such as the study by Walters^29^) to determine the sample size needed for a future trial. Correlations between baseline and follow-up for the continuous outcomes were also estimated, as substantial correlations can be used to reduce the target sample size if linear modelling techniques are used for analysis.

Qualitative interviews were analysed with content analysis^30^ to understand participants’ experiences of key aspects of the trial process and psychological intervention. Key points and supporting quotes are summarised in the results section and Supplementary File 2.

### Missing data

Missing items were imputed using a *pro rata* strategy, provided that at least 75% of the items were available. The exception was the World Health Organization Quality-of-Life Scale (WHOQOL-BREf^31^), where there is a predefined strategy for handling missing data. Please see Supplementary File 3 for details.

## Results

### Participants

Thirty-nine participants were recruited and randomised (see Table 1). The majority were White British (97%, *n* = 38) and female (67%, *n* = 26), with an average age of 67 (s.d. = 6) years. Most (74%, *n* = 29) were in the ‘young old’ age category of 60–69 years.^32^ Most participants were diagnosed with bipolar disorder type 1 (87%, *n* = 34), and average age of diagnosis was 48 (s.d. = 11) years.

**Table 1:** Participant demographic and clinical characteristics

Characteristic	Recovery-focused therapy for older adults with bipolar disorder, *n* = 19	Treatment as usual, *n* = 20
Age, years, mean (s.d.)	66 (5)	68 (6)
Range	60–81	60–81
Gender, *n* (%)		
Male	9 (47)	13 (65)
Female	19 (53)	7 (35)
Marital status, *n* (%)		
Married or living with someone as married	11 (58)	5 (25)
Widowed	2 (11)	4 (4)
Divorced/annulled	6 (32)	7 (35)
Separated	0	1 (5)
Never married	0	3 (15)
Number of children, *n* (%)		
0	2 (11)	4 (20)
1	0	6 (30)
2	12 (63)	5 (25)
3	3 (16)	5 (25)
4	2 (11)	0
Diagnosis		
Bipolar disorder type 1, *n* (%)	15 (79)	19 (95)
Bipolar disorder type 2, *n* (%)	4 (21)	1 (5)
Age of bipolar diagnosis, years, mean (s.d.)	49 (11)	47 (12)
Range	31–80	22–72
On medication, *n* (%)		
Yes	18 (95)	20 (100)
No	1 (5)	0
Number of medications, median	2	2
Range	0–4	1–4
Type of medication, *n* (%)		
Mood stabiliser	15 (79)	13 (65)
Antipsychotic	6 (32)	12 (60)
Antidepressant	8 (42)	6 (30)
Physical health comorbidities, *n* (%)		
Yes	13 (68)	12 (60)
No	5 (26)	7 (35)
Missing	1 (5)	1 (5)
Number of physical health problems, median	2	2
Range	0–6	0–3
Montreal Cognitive Assessment score, mean (s.d.)	26 (2)	25 (3)
Range	22–29	22–30
Previous psychological therapy, *n* (%)		
Never	9 (47)	11 (55)
Cognitive–behavioural therapy	4 (21)	4 (20)
Counselling	1 (5)	4 (20)
Psychotherapy	1 (5)	1 (5)
Dialectical behaviour therapy	1 (5)	0
Psychoeducation group therapy	3 (16)	0

Mean MOCA score was 26 (s.d. = 2), indicating mild to no cognitive impairment.^19^ Most participants reported physical health difficulties (64.1%, *n* = 25), ranging from zero to six difficulties (median of two), consistent with previous research.^3^ Please see Table 1 for further participant demographics and clinical characteristics.

### Participants: qualitative interviews

All participants offered RfT-OA were invited to take part in the qualitative interviews. Eight participants took part: two women and six men, aged 61–72 (mean: 65) years, and attending 7–14 sessions of RfT-OA.

### Key outcomes: feasibility and acceptability

#### Consent, recruitment and retention

**Fig. 1:**
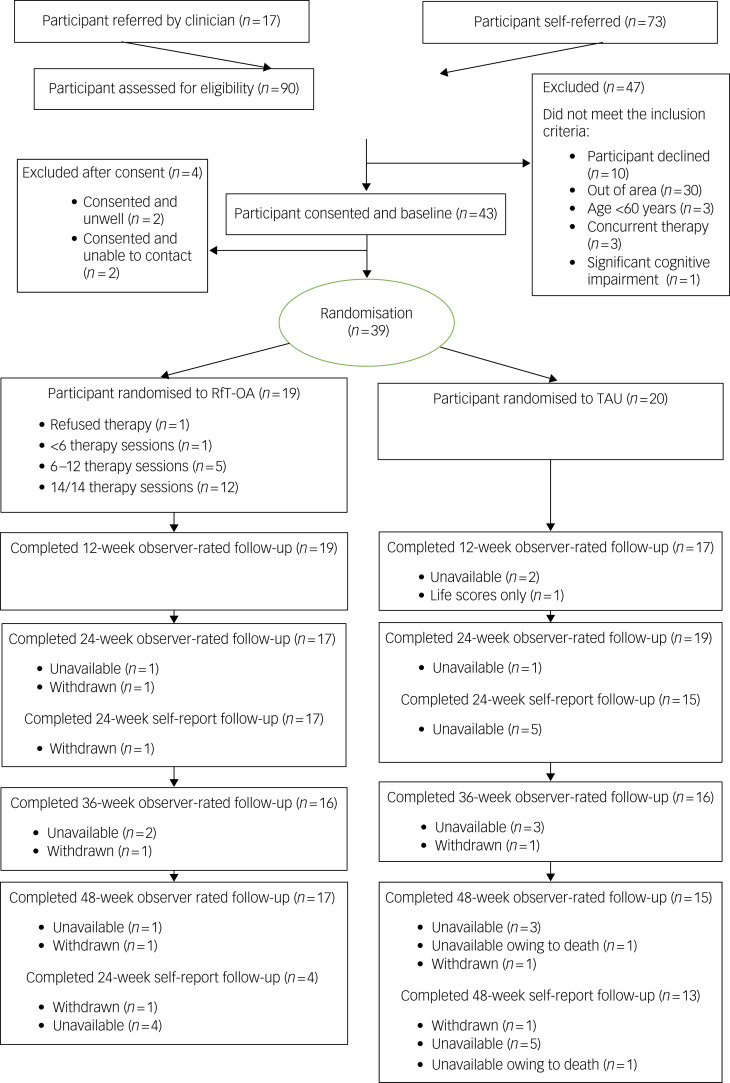
Consolidated Standards of Reporting Trials (CONSORT) diagram. RfT-OA, recovery-focused therapy intervention for bipolar disorder, adapted to meet the needs of older adults; TAU, treatment as usual.

Table 2 summarises feasibility and acceptability outcomes and colour codes with a traffic light system (green, feasible; amber, feasible with modifications; red, stop). Ninety people were referred into the study, 17 by a clinician (Fig. 1). Of the 88 screened for provisional eligibility, 41 did not meet the study criteria, one refused because of physical health difficulties, and three either cancelled or did not attend their baseline interview. Forty-three out of 47 eligible participants consented to participate, although two then became unwell and two consented but then were uncontactable. Thus, 39 out of 47 eligible and consenting participants took part in the trial (green).

**Table 2:** Feasibility and acceptability outcomes

Objective	Measurement process	Feasibility outcome^a^	Final outcome
Estimate the recruitment rate	Recruitment rate is set at a number based on the maximum number of participants that the therapist can see per month. However, number of eligible participants recruited will be recorded on a monthly basis, to inform the recruitment plan for a larger trial	Green: Feasibility will be shown where 3–4 participants are recruited per month over the 15-month recruitment window (approximately *n* = 50). Amber: If two or more participants are recruited per month (approximately 30 participants) or four to five participants are recruited in the past 6 months of the trial (if recruitment problems are overcome), then a future trial will be feasible but additional strategies will be identified to achieve target recruitment. Red: If fewer than two participants are recruited per month (*n* <25 total participants) over the recruitment period, feasibility will not be demonstrated	39 participants recruited over 17 months. Average of 2.3 participants per month, therefore strategies to overcome potential barriers will be identified
Identify consent rate and reasons for non-recruitment	Number of referred participants that are eligible but choose not to consent to the trial will be recorded, and reasons for refusal will be documented where offered	Green: Feasibility will be shown if ≥80% of eligible participants referred provide consent to the trial. Amber: If 60–79% of eligible participants referred provide consent to the trial, then a future trial will be feasible if strategies to overcome identified barriers are identified (including whether more individuals are consenting who self-refer or are referred by clinicians). Red: If <60% of eligible participants referred do not consent to the trial, then feasibility will not be demonstrated	39 out of 47 eligible participants consented to the trial. The consent rate is 83%, therefore feasibility is demonstrated
Estimate the proportion of participants lost to follow-up and the reasons for loss to follow-up	The loss of participants during the follow-up period will be recorded, plus reasons for loss (if given)	Green: Feasibility will be demonstrated if ≥70% of participants are retained at the 48-week follow-up. Amber: If 50–69% of participants are retained at the 48-week follow-up, then a future trial will be feasible if strategies to overcome identified barriers are identified.	32 out of 39 participants completed the observer-rated measures at 48 weeks. The retention rate is 82%, therefore feasibility is demonstrated.
		Red: If <50% of participants referred do not consent to the trial, then feasibility will not be demonstrated	27 out of 39 participants returned their self-report measures at 48 weeks. The retention rate is 69%, therefore strategies to overcome potential barriers will be identified
Estimate the number of therapy sessions attended	The number of therapy sessions attended out of the 14 offered will be recorded	Green: Feasibility will be demonstrated if all of the participants attend six or more sessions^a^ of the 14 offered. Amber: If 75–99% of participants attend six or more sessions of the 14 offered, a future trial will be feasible if strategies to overcome barriers are identified. Red: If <75% of participants do not attend six or more of the therapy sessions offered, then feasibility will not be demonstrated	17 out of 19 participants attended six or more sessions. Attendance rate for six or more sessions is 89%, therefore strategies to overcome potential barriers will be identified
Estimate the number of participants who drop out of therapy	The number of participants who drop out of the therapy sessions will be recorded	Green: If ≥65% of the participants in the intervention arm complete therapy, then feasibility will be demonstrated. Amber: If 50–64% of participants in the intervention arm complete therapy, then a future trial will be feasible if strategies to overcome drop-out are identified. Red: If <50% of participants in the intervention arm drop out of therapy, then feasibility will not be demonstrated	13 out of 19 participants attended all 14 sessions. Completeness rate for therapy is 68%, therefore feasibility has been demonstrated
Assess the feasibility of delivering recovery-focused therapy in a way that is acceptable to people with bipolar disorder in later life	Interviews with 10–15 participants that have taken part in the intervention arm of the study, to seek their views on the therapy	Feasibility will be demonstrated if >50% of participants indicate that the intervention is acceptable	>50% of participants indicated they valued the intervention. The majority of participants felt that the session number, length and location were acceptable

a. Criteria were developed by the research team (E.T., S.H.J., F.L. and C.S.), based on previous trials for individuals with bipolar disorder^10,33^ and comparable studies investigating psychotherapeutic treatment for depression in later life.^34,35^

Thirty-nine participants were randomised (29 via self-referral and ten identified by a clinician) over 17 months; two participants per month (amber).

Retention for observer-rated measures was over 80% for each follow-up, with 82% (*n* = 32) retention at 48-week follow-up (green). Retention for the self-report measures was over 80% at the 24-week follow-up and 69% (*n* = 27) for the 48-week follow-up self-report (amber). One participant lost to follow-up died during the final follow-up period for reasons unrelated to the trial, as confirmed by the trial steering committee.

Eight baseline interviews were double-rated by D.D. to assess whether participants met study criteria (age, MOCA score, a bipolar disorder type 1 or 2 diagnosis and to confirm the participant was not in a current episode). Fifteen follow-up interviews were also double-rated to assess whether the participants met criteria for an SCID-5-RV episode and type of episode during a follow-up period. There was 100% agreement regarding eligibility criteria and for follow-up interviews.

#### Therapy retention

Mean attendance was 12.2 (s.d. = 3.3) sessions. One participant did not start therapy, 89% (*n* = 17) attended more than six sessions (amber) and 68% (*n* = 13) attended all 14 sessions (green).

### Additional outcomes: feasibility and acceptability

#### Therapeutic alliance

Alliance data was available from 15 clients at the start of therapy (mean age 63 years, s.d. = 5), eight clients in the middle of therapy (mean age 66 years, s.d. = 5) and nine clients at the end of therapy (mean 65 years, s.d. = 7). Alliance rated by the therapists was similar, with 15 ratings at the start of therapy (mean age 64 years, s.d. = 6), eight ratings in the middle of therapy (mean age 63 years, s.d. = 7) and nine ratings at the end of therapy (mean age 65 years, s.d. = 6). Alliance ratings are similar to those observed for psychological therapy in cohorts of younger people with bipolar disorder.^10,33^

#### Client ratings of therapy

Data was available for 15 participants: the usefulness of therapy score average was 9 (s.d. = 2) and the likelihood of recommending therapy score average was 9 (s.d. = 1), meaning that participants found therapy very useful and were very likely to recommend to a friend.

### Qualitative interviews findings

#### Participants’ experiences of the intervention

The majority of participants indicated that they valued RfT-OA and highlighted its positive impact on their lifestyle, family and work relationships. One participant found it difficult to engage because of concurrent marital problems. Value was derived from both the recovery approach and learning strategies to manage emotions in a new way. The majority of participants felt that 14 therapy sessions was enough. The participant who had not felt the benefit from the sessions attended seven sessions and expressed that they would have liked more. Participants felt the session length of 50–60 min was sufficient, and valued the flexibility of the sessions being offered at home, work or the university. See Supplementary File 4 for example quotes.

#### Research process

On the whole, participants found the research process acceptable, although five out of eight participants indicated they would prefer the follow-up appointments to be face to face rather than on the phone. Participants were positive about receiving and returning the self-report measures by post. Participants did not express strong views on the acceptability and relevance of individual outcome measures. See Supplementary File 4 for example quotes.

### Clinical and functional measures

#### Effect size estimation for candidate primary outcome measures

##### BRQ

BRQ scores in both groups were increased at the 24-week follow-up, which was numerically sustained in the intervention group and reduced in the TAU at 48 weeks (Table 3). However, there is not a clear signal of potential clinical effectiveness. Pooled s.d. was 369.4 and 359.2 at 24 and 48 weeks, respectively. The estimated correlation between the baseline and the 24-week scores was high (0.73), but there was no positive correlation between baseline and 48-week scores.

**Table 3:** Candidate primary outcome measures

	TAU, *n*	TAU, mean	TAU, s.d.	RfT-OA, *n*	RfT-OA, mean	RfT-OA, s.d.	Mean difference (adjusted 95% CI)
Bipolar Recovery Questionnaire							
Baseline	19	2026.2	316.8	18	1914.8	352.2	
24-week follow-up	14	2248.0	359.8	16	2134.9	377.5	22.5 (–172.4 to 217.3)
48-week follow-up	12	2075.2	374.8	14	2136.2	345.3	62.9 (–238.3 to 364.0)
HRSD							
Baseline	20	5.0	4.5	19	4.5	4.0	
24-week follow-up	19	9.7	8.7	17	5.3	4.5	–4.2 (–8.9 to 0.5)
48-week follow-up	15	9.4	10.30	17	4.8	4.1	–4.2 (–9.8 to 1.4)
MAS							
Baseline	20	2.2	3.1	19	1.8	2.7	
24-week follow-up	19	3.9	5.2	17	1.7	2.2	–1.8 (–4.3 to 0.7)
48-week follow-up	15	2.6	2.9	17	1.0	1.5	–1.8 (–3.4 to –0.1)
	*χ*^2^	d.f.	Hazard ratio	Lower 95% confidence limit	Upper 95% confidence limit		
Time to any relapse	10.1	3	0.23	0.07	0.73		
Time to manic relapse	6.1	3	0.44	0.13	1.51		
Time to depressive relapse	7.7	3	0.13	0.01	1.06		

See Supplementary File 4 for *P*-values. TAU, treatment as usual; RfT-OA, recovery-focused therapy for older adults with bipolar disorder; HRSD, Hamilton Rating Scale for Depression; MAS, Bech–Rafaelsen Mania Scale.

##### SCID-LIFE (time-to-relapse)

RfT-OA participants had fewer depressive or manic relapses (18 TAU *v*. 7 RfT-OA), and demonstrated a longer median time to relapse (15.5 *v*. 8.5 weeks) during follow-up.

Over 48 weeks, 17 participants experienced a depressive relapse (11 TAU *v*. 6 RfT-OA) and eight experienced a manic episode (7 TAU *v*. 1 RfT-OA).

##### HRSD

HRSD scores indicated mild depression on average in TAU at 24- and 48-week follow-up, compared with no depression in RfT-OA (Table 3). The pooled s.d. was 7.0 at 24 weeks and 7.6 at 48 weeks. The estimated correlation between the baseline and follow-up scores was very low (0.15 at 24 weeks and 0.04 at 48 weeks).

##### MAS

MAS scores remained low throughout, indicating low levels of mania (Table 3). MAS score was lower in the RfT-OA group at both the 24- and 48-week follow-up compared with TAU, suggesting lower levels of mania following therapy. The pooled standard deviation was 4.1 at 24 weeks and 2.2 at 48 weeks. The estimated correlation between the baseline and 24-week scores was 0.44, but there was no positive correlation between baseline and 48-week scores.

#### Completion rates for candidate primary outcome measures

##### BRQ

At baseline, 39 out of 39 participants completed the BRQ, although two questionnaires were missing ≥25% of the data; therefore 37 questionnaires were included. At 24 weeks, 31 out of 39 questionnaires were returned, with one excluded from analyses because of missing item data. At 48 weeks, 27 out of 39 questionnaires were returned, with one excluded from analyses because of missing data.

##### Time to relapse, HRSD and MAS

Observer-rated assessments measuring time to relapse and mood symptoms were completed at baseline, 12, 24, 36 and 48 weeks. Completion rates were 36 out of 39 participants at 24 weeks, and 32 out of 39 participants at 48 weeks, because of either participant drop-out or unavailability.

#### Acceptability of candidate primary outcome measures

Participants did not give any specific feedback about particular questionnaires, although they indicated that the questionnaires in general were easy to understand (see Supplementary File 4).

#### Additional clinical outcomes

Additional clinical outcomes are reported in Supplementary File 5, with higher mean scores observed in the Personal and Social Performance Scale in RfT-OA at both 24- and 48-week follow-up, indicating higher levels of functioning. The other clinical outcome measures tended to favour RfT-OA, although this was not consistent across all measures and time points.

## Discussion

This is the first study to develop and evaluate a psychological intervention specifically for older adults with bipolar disorder, using a randomised controlled trial design. RfT-OA was developed and designed in collaboration with individuals living with bipolar disorder,^21^ enhancing the quality, value and relevance of the study.

### Feasibility and acceptability of RfT-OA

The study findings largely support the feasibility and acceptability of RfT-OA, evaluated against predefined criteria. It was possible to recruit 39 participants at a rate of 2.3 per month (amber progression zone). This was with the lead researcher as the sole recruiter, working part-time for the second half of the recruitment period. A well-resourced research team would be in a stronger position to enhance the recruitment rate (discussed further below). Retention to follow-up was strong and balanced across trial arms, with rates comparing favourably to previous bipolar disorder and older adult depression trials.^10,15,36^ Intervention arm participants engaged with RfT-OA, demonstrating a significant commitment to therapy. Two participants did not attend six or more therapy sessions (amber); therefore, strategies to overcome any potential barriers are discussed below. Interviews indicated that participants valued RfT-OA, corresponding with high client ratings of therapy usefulness and recommending to a friend. Alliance ratings were acceptable and comparable to younger bipolar disorder cohorts.^10,33^

### Primary outcome measure

Completion rates were higher for the observer-rated measures (e.g. time to relapse and mood symptoms; 86–90%) than the self-report measures (e.g. the BRQ; 70–81%), although a number of strategies have been identified below to enhance self-report rates. Feedback from the interviews indicated that participants were positive about the data collection process and did not report any difficulties completing any of the measures. The MAS, HRSD and SCID-LIFE (time to relapse) demonstrated a signal of benefit, although the trial was not powered to test intervention effectiveness. Further consultation with older adults with bipolar disorder will be needed to identify which measure is most relevant to their experiences and what they would want to change during RfT-OA, before confirming the most appropriate and meaningful primary outcome measure for a definitive trial.

### Sample size for a future trial

To help determine the appropriate sample size for a future trial, pooled standard deviations for MAS and HRSD and the median time to relapse were estimated. For the HRSD, a minimal clinically important difference in a trial context is often deemed to be around 3 points,^37^ with an s.d. of 7–8 points (consistent with our results). Using a conservative 8-point s.d. (standardised effect size of 0.375), a sample size of 302 would be needed to achieve 90% power (two-sided 5% significance level using a two-sample *t*-test), inflated to 404 (202 per group) to allow for an anticipated 25% attrition. Baseline-outcome correlations were also estimated. These, however, were generally low; only that for the 24-week MAS score was sufficiently large to enable a useful reduction in target sample size. For both the HRSD and MAS, average scores were low throughout, which may bring into question the appropriateness of these as primary outcome measures. The estimated median time to relapse in TAU was 8.5 weeks, which is somewhat lower than previous research on a similar population (14 weeks^38^). Using a conservative median time to relapse of 18 weeks, if participants were followed up for relapse for 52 weeks (SCID-LIFE data with data from case notes where needed), using a Cox regression model (with 5% significance level), 320 participants would be needed to achieve 90% power to detect a hazard ratio of 2/3.

### Limitations

There are a number of limitations to the study. First, information on what constituted TAU was limited. Referral route, medication use and information on previous psychological therapy were collected to help define this; however, it would be useful to collect information regarding current level of care for a future trial. Second, it was not feasible to employ a blinded research assistant to carry out the assessment and follow-up. Third, data from people who had few or no sessions of RfT-OA were lacking for the qualitative interviews. Finally, this was a relatively small study, conducted in North-West England, with a predominantly White British sample. A definitive trial would need to recruit a more geographically and ethnically diverse sample to support generalisability of findings.

### Lessons for a future trial

There was substantially more interest from the self-referral recruitment route compared with the clinical route. A well-resourced research team would be poised to form stronger links with clinical teams, generating further interest and increasing the recruitment rate through both routes. A number of strategies have been identified from recent research^39^ to enhance completion rates for self-report measures, including providing detailed explanations regarding the importance of data completeness in recruitment materials, collecting multiple contact details and reminders via different methods (e.g. telephone, text, post, email). Preference for a face-to-face interview for follow-up was highlighted in the qualitative interviews, therefore face to face, video or audio call could be offered to support completion of self-report measures. Paying participants for completing the measures could further enhance retention and is considered good practice with the National Institute for Health Research. Additionally, the observer measures may have been affected by bias. A definitive trial would be fully costed to employ independent, blind assessors to avoid any risks of bias. Finally, two participants did not attend six sessions of therapy: one person became unwell and did not stabilise before the end of the 6-month therapy window, and one person dropped out because of personal reasons. In a future trial, the option of extending the therapy window will be considered with a view to re-engaging participants who disengage with therapy during the trial.

Despite these limitations, the trial was successful in demonstrating the feasibility and acceptability of recruitment, retention and intervention processes. The majority of participants started therapy when offered and adhered to the intervention, reporting positive benefits. The assessment measures provide evidence for a signal for effectiveness on a range of outcomes, including mood symptoms, time to relapse and functioning. The impact on time to relapse, in particular, suggests that RfT-OA may be linked to reduced service costs; however, this would require formal evaluation in a definitive trial. A definitive trial is now warranted to provide a robust estimate of the clinical and cost effectiveness of RfT-OA, and to provide an important step for a group of individuals who, at present, do not have access to evidence-based psychological care.

## Data Availability

The data that support the findings of this study are available from the corresponding author, E.T., upon reasonable request. The transcripts from the qualitative interviews cannot be published or made available if requested, to protect the anonymity of the participants who took part.

## Supplementary material

For supplementary material accompanying this paper visit http://doi.org/10.1192/bjo.2022.582. click here to view supplementary material
