# Supplementary material for: A pilot randomised controlled trial to assess the feasibility and acceptability of recovery-focused therapy for older adults with bipolar disorder
Source: BJPsych Open. 2022 Oct 24;8(6):e191. doi: 10.1192/bjo.2022.582 (PMC9634560; doi:10.1192/bjo.2022.582)
Supplement: Supplementary file 1 [file S2056472422005828sup001.zip › S2056472422005828sup002.docx]

**Supplementary file 5**

**Observer rated measures**

Personal and Social Performance Scale (PSP; 1)

Structured Clinical Interview for DSM-5, research version (SCID-5-RV; 2)

Hamilton Rating Scale for Depression (HRSD; 3)

The modified Longitudinal Interview Follow-up (SCID-LIFE; 4)

**Self-report measures**

Bipolar Recovery Questionnaire (BRQ; 5),

Quality of Life in Bipolar Disorder Scale (QoL.BD; 6),

The Internal State Scale (ISS; 7),

The Centre for Epidemiologic Studies Depression Scale (CES-D; 8),

The Work and Social Adjustment Scale (WSAS; 9)

The WHO Quality of Life Scale (WHOQOL-BREf; 10).

**References**

1. Morosini P, Magliano L, Brambilla L, Ugolini S, Pioli R. Development, reliability and acceptability of a new version of the DSMIV social and occupational functioning assessment scale (SOFAS) to assess routine social functioning. Acta Psychiatr Scand. 2000; 101: 323–9.

2. First MB, Williams JBW, Karg RS, Spitzer RL. Structured Clinical Interview for DSM-5—Research Version (SCID-5 for DSM-5, Research Version; SCID-5-RV). Arlington, VA, American Psychiatric Association, 2015.

3. Hamilton M. A rating scale for depression. J Neurol Psychiatry 1960; 23: 59–62.

4. Bech P, Rafaelsen OJ, Kramp P, Bolwig TG. The mania rating scale: scale construction and inter-observer agreement. Neuropharmacol. 1978; 17: 430–1.

5. Keller MB, Lavori PW, Friedman B, Nielsen E, Endicott J, McDonald-Scott P, Andreasen NC. The longitudinal interval follow-up evaluation. a comprehensive method for assessing outcome in prospective longitudinal studies. Arch Gen Psychiatry. 1987; 44: 540–8.

6. Jones S, Mulligan LD, Higginson S, Dunn G, Morrison A. The bipolar recovery questionnaire: psychometric properties of a quantitative measure of recovery experiences in bipolar disorder. J Affect Disord. 2013; 147: 34–43.

7. EE, Murray G, Crest BD. Development of the QoL.BD: a disorder specific scale to assess quality of life in bipolar disorder. Bipolar Disord. 2010; 12: 727–40.

8. Bauer MS, Crits-Christoph P, Ball WA, Dewees E, McAllister T, Alahi P, Cacciola J, Whybrow PC. Independent assessment of manic and depressive symptoms by self-rating. Scale characteristics and implications for the study of mania. Arch Gen Psychiatry. 1991; 48: 807–12.

9. Radloff LS. The CES-D scale: a self-report depression scale for research in the general population. Appl Psychol Meas. 1977; 1:385–401.

10. Mundt JC, Marks IM, Shear MK, Greist JH. The Work and Social Adjustment Scale: a simple measure of impairment in functioning. Br J Psychiatry. 2002; 180:461–4.

11. The WHOQOL Group. Development of the World Health Organization WHOQOL-BREF quality of life assessment. Psychol Med. 1998; 28(3): 551-558.
