# Supplementary material for: A pilot randomised controlled trial to assess the feasibility and acceptability of recovery-focused therapy for older adults with bipolar disorder
Source: BJPsych Open. 2022 Oct 24;8(6):e191. doi: 10.1192/bjo.2022.582 (PMC9634560; doi:10.1192/bjo.2022.582)

**Supplementary file 3**

**Scoring the WHOQOL-BREF**

The WHOQOL-BREF (Field Trial Version) produces a quality of life profile. It is possible to derive four domain scores. There are also two items that are examined separately: question 1 asks about an individuals overall perception of quality of life and question 2 asks about an individuals overall perception of their health. The four domain scores denote an individuals perception of quality of life in each particular domain. Domain scores are scaled in a positive direction (i.e. higher scores denote higher quality of life).

The mean score of items within each domain is used to calculate the domain score. Mean scores are then multiplied by 4 in order to make domain scores comparable with the scores used in the WHOQOL-100. Explicit instructions for checking and cleaning data, and for computing domain scores, are given in Table 3. A method for the manual calculation of individual scores is given on page 1 of the WHOQOL-BREF assessment form. The method for converting raw scores to transformed scores when using this method is given in Table 4, on page 11 of these instructions. The first transformation method converts scores to range between 4-20, comparable with the WHOQOL-100. The second transformation method converts domain scores to a 0-100 scale.

Where more than 20% of data is missing from an assessment, the assessment should be discarded (see Step 4 in Table 3). Where an item is missing, the mean of other items in the domain is substituted. Where more than two items are missing from the domain, the domain score should not be calculated (with the exception of domain 3, where the domain should only be calculated if < 1 item is missing). Any national items should be scored separately from the core 26 item of the BREF. During the analysis the performance of any national items will be examined for possible use in alter national studies. At this stage of field testing national and core items must not be mixed in administration or scoring of the BREF.


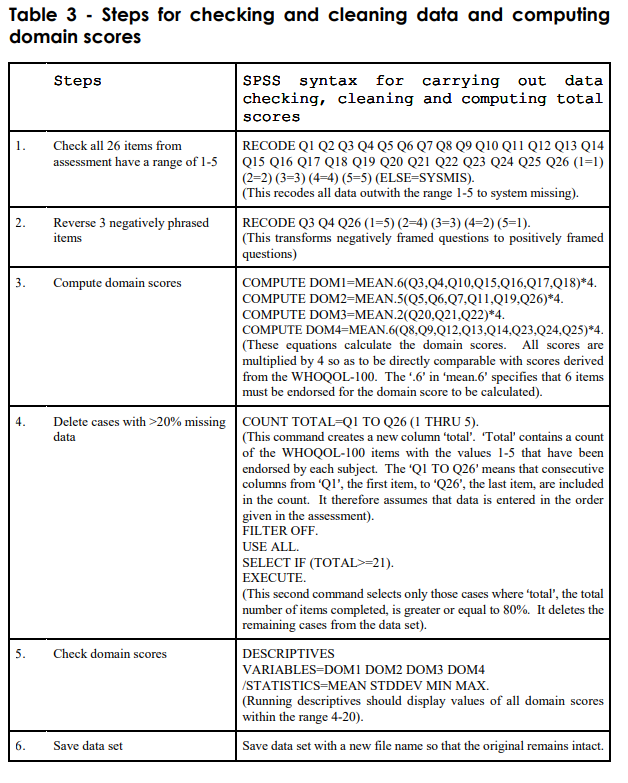

Supplement: Supplementary file 1 [file S2056472422005828sup001.zip › S2056472422005828sup003.docx]
