# Supplementary material for: A pilot randomised controlled trial to assess the feasibility and acceptability of recovery-focused therapy for older adults with bipolar disorder
Source: BJPsych Open. 2022 Oct 24;8(6):e191. doi: 10.1192/bjo.2022.582 (PMC9634560; doi:10.1192/bjo.2022.582)
Supplement: Supplementary file 1 [file S2056472422005828sup001.zip › S2056472422005828sup004.docx]

**Supplementary file 2**

| Experience of intervention | |
| --- | --- |
| Positive impact on the lifestyle | *“A greater focus on what my lifestyle’s like and what I’m doing and when I’m doing it…looking at sleep pattern, getting it undisturbed sleep now, so that was useful” (P002)* |
| Positive impact on relationships | *“I’m more relaxed around them, and they’re more relaxed around me” (P003)*  *“I’ve learnt to be perhaps more patient really with other people, um, and not be demanding of them… I still do rely on my husband a lot but I try not to over rely on him and expect him to do things that are too perfectionist” (P005)* |
| Recovery focused approach | *I thought it was well balanced and aimed at specific elements of your mental health er process of recovery …it’s like giving out a silver bullet, that bullet that drives right to the centre of the problems (P003)* |
| Coping strategies to manage mood | *I think er if I feel down, I think um, I can… give myself permission to be down, er but be aware not to let it get into a spiral and do…somethings which are soothing, some things which are stimulating, you’ve got to make your choice at the time (P005)* |
| Difficulty engaging in therapy | *Not a lot has changed – we were going through marital problems at the time. We were too busy trying to sort ourselves out (P007)* |
| Therapist stance | *There were lots of intimate things we discussed…I didn’t feel that there was any judgement there (P001)* |
| Session number | *“I think it was about right, ‘cos you needed a couple of sessions just to get going and get to know what’s happening and what the strategy is going to be” (P002)*  *“I was a bit disappointed when it ended really but I recognise that you know it couldn’t go on forever” (P001)*  *“Only did 7 sessions – should have had more as it was not enough”* |
| Session location | *“I thought it was excellent, I couldn’t believe it really when it was offered (to have the therapy sessions at home; P001)*  *“I enjoyed it coming to work… because it reinforced when I said to people look XX is coming to work and we’re going into this room … it reinforced to them my mental health conditions” P003* |
| Session timing (50-60 minutes) | *“It’s very flexible, so it works, no longer though” (P003)*  *“You can keep on track better over an hour...some people start going round in circles in less time… but I think an hour is about right” P005* |
| Research process | |
| Follow-up assessments | *“I prefer face to face any time” (P006)*  *“It was ok on the phone but obviously face to face is better” (P001)* |
| Questionnaire by post | *“That was alright by post, that was ok” (P008)*  *“online you can unclick them can’t you if you think I’ve read that wrong...so it’s easier to unclick and make it clear rather than think when you’ve ticked a box and scribbled it out” (P005)* |
| Completing the questionnaires | *“I filled in all the various forms that came” (P007)*  *“questionnaires were fine, they were easy to understand and easy to get through” P005*  *“I thought they were simple, even though it was a lot, it was easy to go through and none of it was challenging” (P003)* |
