# Supplementary material for: A pilot randomised controlled trial to assess the feasibility and acceptability of recovery-focused therapy for older adults with bipolar disorder
Source: BJPsych Open. 2022 Oct 24;8(6):e191. doi: 10.1192/bjo.2022.582 (PMC9634560; doi:10.1192/bjo.2022.582)
Supplement: Supplementary file 1 [file S2056472422005828sup001.zip › S2056472422005828sup005.docx]

**Supplementary file 4**

|  | TAU  N | TAU  Mean | TAU  s.d. | RfCBT-OA  N | RfCBT-OA  Mean | RfCBT-OA  s.d. |
| --- | --- | --- | --- | --- | --- | --- |
| **QoL-BD**  Baseline  24 week follow-up  48 week follow-up | 20  15  12 | 42.40  41.44  38.41 | 11.61  12.10  12.38 | 19  17  14 | 38.68  40.98  39.07 | 7.21  9.25  7.97 |
| **CES-D**  Baseline  24 week follow-up  48 week follow-up | 20  14  13 | 21.85  18.50  21.10 | 12.42  9.33  11.27 | 19  16  13 | 23.66  17.31  21.85 | 9.20  7.63  9.34 |
| **Internal States Scale - Activation**  Baseline  24 week follow-up  48 week follow-up | 18  14  11 | 178.33  137.86  133.64 | 119.82  97.99  100.33 | 19  17  13 | 95.26  93.53  47.69 | 100.41  104.88  65.47 |
| **Internal States Scale - Wellbeing**  Baseline  24 week follow-up  48 week follow-up | 19  14  11 | 182.11  181.43  193.64 | 83.77  88.74  84.06 | 19  17  13 | 118.95  143.53  137.69 | 74.23  100.81  67.35 |
| **WASAS**  Baseline  24 week follow-up  48 week follow-up | 20  15  8 | 19.69  14.88  17.38 | 13.17  12.97  13.99 | 18  17  13 | 19.83  16.31  14.77 | 10.58  10.49  6.67 |
| **WHO-QOL BREF – Physical**  Baseline  24 week follow-up  48 week follow-up | 19  13  12 | 56.05  63.05  57.44 | 21.22  24.79  19.99 | 19  17  14 | 53.98  57.35  56.51 | 15.50  19.43  16.02 |
| **WHO-QOL BREF – Psychological**  Baseline  24 week follow-up  48 week follow-up | 19  12  12 | 58.77  68.75  62.99 | 21.66  14.38  21.36 | 19  17  14 | 52.19  56.62  56.25 | 20.24  19.38  19.99 |
| **WHO-QOL BREF – Social**  Baseline  24 week follow-up  48 week follow-up | 19  12  12 | 53.29  61.11  56.94 | 24.32  29.96  22.71 | 19  16  13 | 58.33  58.85  53.21 | 19.64  21.83  24.89 |
| **WHO-QOL BREF – Environmental**  Baseline  24 week follow-up  48 week follow-up | 19  13  12 | 67.11  75.49  69.38 | 24.72  16.77  14.29 | 19  17  14 | 72.89  69.41  68.75 | 14.52  15.30  14.91 |
| **Personal and Social Performance Scale**  Baseline   24 week follow-up  48 week follow-up | 20  19  15 | 71.90  68.58  71.07 | 10.68  12.90  14.46 | 19  17  17 | 75.42  77.06  76.71 | 10.02  8.89  11.09 |

**Linear Models**

Bipolar Recovery Questionnaire

| RfCBT vs TAU | MD; 95% CI | P-value |
| --- | --- | --- |
| 24 week | 22.471 (-172.446 to 217.387) | 0.815 |
| 48 week | 62.852 (-238.257 to 363.960) | 0.670 |

Hamilton Rating Scale for Depression

| RfCBT vs TAU | MD; 95% CI | P-value |
| --- | --- | --- |
| 24 week | -4.207 (-8.949 to 0.536) | 0.080 |
| 48 week | -4.206 (-9.780 to 1.368) | 0.134 |

Bech-Rafaelsen Mania Scale

| RfCBT vs TAU | MD; 95% CI | P-value |
| --- | --- | --- |
| 24 week | -1.765 (-4.257 to -0.727) | 0.159 |
| 48 week | -1.792 (-3.464 to -0.119) | 0.037 |

Quality of Life in Bipolar Disorder Scale

| RfCBT vs TAU | MD; 95% CI | P-value |
| --- | --- | --- |
| 24 week | 3.145 (-2.991 to 9.281) | 0.303 |
| 48 week | 3.091 (-4.930 to 11.111) | 0.434 |

Centre for Epidemiologic Studies Depression Scale

| RfCBT vs TAU | MD; 95% CI | P-value |
| --- | --- | --- |
| 24 week | -4.184 (-9.301 to 0.933) | 0.105 |
| 48 week | -0.222(-8.217to 7.773) | 0.955 |

Internal States Scale - Activation

| RfCBT vs TAU | MD; 95% CI | P-value |
| --- | --- | --- |
| 24 week | -14.983 (-81.288 to 51.323) | 0.647 |
| 48 week | -61.100 (-133.209 to 11.009) | 0.092 |

Internal States Scale - Well-being

| RfCBT vs TAU | MD; 95% CI | P-value |
| --- | --- | --- |
| 24 week | 34.832 (-27.774 to 97.438) | 0.264 |
| 48 week | -34.143 (-106.724 to 38.437) | 0.339 |

Work and Social Adjustment Scale

| RfCBT vs TAU | MD; 95% CI | P-value |
| --- | --- | --- |
| 24 week | -1.095 (-6.488 to 4.298) | 0.681 |
| 48 week | -3.778 (-10.445 to 2.888) | 0.248 |

WHO-QOL BREF – Physical

| RfCBT vs TAU | MD; 95% CI | P-value |
| --- | --- | --- |
| 24 week | -10.028 (-25.596 to 5.540) | 0.197 |
| 48 week | -5.543 (-19.694 to 8.608) | 0.426 |

WHO-QOL BREF - Psychological

| RfCBT vs TAU | MD; 95% CI | P-value |
| --- | --- | --- |
| 24 week | -3.592 (-14.352 to 7.168) | 0.498 |
| 48 week | 2.02 (-11.92 to 15.95) | 0.767 |

WHO-QOL BREF – Social

| RfCBT vs TAU | MD; 95% CI | P-value |
| --- | --- | --- |
| 24 week | 3.123 (-13.450 to 19.703) | 0.701 |
| 48 week | -2.597 (-16.284 to 11.091) | 0.697 |

WHO-QOL BREF – Environmental

| RfCBT vs TAU | MD; 95% CI | P-value |
| --- | --- | --- |
| 24 week | -2.559 (-9.882 to 4.765) | 0.479 |
| 48 week | -1.846 (-10.141 to 6.450) | 0.649 |

Personal and Social Performance Scale

| RfCBT vs TAU | MD; 95% CI | P-value |
| --- | --- | --- |
| 24 week | 5.786 (1.320 to 10.251) | 0.013 |
| 48 week | 2.644 (-3.518 to 8.806) | 0.387 |

Key: CI = Confidence interval, MD= Mean difference

**Cox Regression**

Time to relapse - Any relapse

|  | **χ2** | **Df** | **Exp(B)** | **Lower 95% CL** | **Upper 95% CL** | **P-Value** |
| --- | --- | --- | --- | --- | --- | --- |
| Time to any relapse | 10.1 | 3 | 0.23 | 0.07 | 0.73 | 0.013 |

Time to relapse - Depression

|  | **χ2** | **Df** | **Exp(B)** | **Lower 95% CL** | **Upper 95% CL** | **P-Value** |
| --- | --- | --- | --- | --- | --- | --- |
| Time to depressive relapse | 7.7 | 3 | 0.13 | 0.01 | 1.06 | 0.057 |

Time to relapse - Mania

|  | **χ2** | **Df** | **Exp(B)** | **Lower 95% CL** | **Upper 95% CL** | **P-Value** |
| --- | --- | --- | --- | --- | --- | --- |
| Time to manic relapse | 6.1 | 3 | 0.44 | 0.13 | 1.51 | 0.195 |

Key: χ2 = Chi Squared, Df = Degrees of freedom , CL = Confidence limit
